# Silage preparation and sustainable livestock production of natural woody plant

**DOI:** 10.3389/fpls.2023.1253178

**Published:** 2023-09-08

**Authors:** Zhumei Du, Fuyu Yang, Jiachen Fang, Seishi Yamasaki, Tetsuji Oya, Damiao Nguluve, Hajime Kumagai, Yimin Cai

**Affiliations:** ^1^ College of Animal Science and Technology, Yangzhou University, Yangzhou, China; ^2^ Crop, Livestock, and Environment Division, Japan International Research Center for Agricultural Sciences (JIRCAS), Tsukuba, Ibaraki, Japan; ^3^ College of Animal Science, Guizhou University, Guiyang, China; ^4^ Faculty of Agriculture and Life Science, Hirosaki University, Hirosaki, Japan; ^5^ Animal Science Directorate, Agricultural Research Institute of Mozambique, Matola, Mozambique; ^6^ Graduate School of Agriculture, Kyoto University, Kyoto, Japan

**Keywords:** animal product, natural biomass resource, silage fermentation, sustainable livestock production, woody plant

## Abstract

As the global population increases and the economy grows rapidly, the demand for livestock products such as meat, egg and milk continue to increase. The shortage of feed in livestock production is a worldwide problem restricting the development of the animal industry. Natural woody plants are widely distributed and have a huge biomass yield. The fresh leaves and branches of some woody plants are rich in nutrients such as proteins, amino acids, vitamins and minerals and can be used to produce storage feed such as silage for livestock. Therefore, the development and utilization of natural woody plants for clean fermented feed is important for the sustainable production of livestock product. This paper presents a comprehensive review of the research progress, current status and development prospects of forageable natural woody plant feed resources. The nutritional composition and uses of natural woody plants, the main factors affecting the fermentation of woody plant silage and the interaction mechanism between microbial co-occurrence network and secondary metabolite are reviewed. Various preparation technologies for clean fermentation of woody plant silage were summarized comprehensively, which provided a sustainable production mode for improving the production efficiency of livestock and producing high-quality livestock product. Therefore, woody plants play an increasingly important role as a potential natural feed resource in alleviating feed shortage and promoting sustainable development of livestock product.

## Highlights

- A review of recent advances in the renewable use of natural woody plant.
- Natural woody plant produces clean feed to alleviate feed shortage.
- Fermented feed of woody plant produces livestock product with added value.
- Woody plant contributes to the sustainable production of livestock product.

## 1 Introduction

With the rapid development of the global economy, per capita consumption levels and demand for livestock products such as meat, eggs and milk are increasing (Komarek et al., 2021). Along with the development of livestock farming, the demand for quality forage is also increasing (Keeling et al., 2019). Currently, the main factor affecting livestock production is the insufficient supply of livestock forage, which in many countries is derived from feed crops, grasses, crop by-products and cereals (Cai et al., 2020a; Cai et al., 2021). With increasing global population and decreasing per capita arable land, traditional forage production methods cannot meet the demand for livestock feeding, leading to feed shortages and affecting the sustainable production of livestock products worldwide (Du et al., 2021b). Therefore, there is an urgent need to develop new feed resources, such as nutrient-rich natural woody plant resources, to meet the challenges posed by the rapid development of the livestock industry (Owen-Smith and Cooper, 1987; Vandermeulen et al., 2017).

The major woody plant available for feed worldwide are mulberry [*Morus alba* (L.)], moringa [*Moringa oleifera* (L.)], gliricidia [*Gliricidia sepium* (Jacq.)], leucaena [*Kunth ex Walp. leucocephala* (L.) de Wit], and paper mulberry [*Broussonetia papyrifera* (L.)]. These woody plants are deciduous trees or shrubs that are highly adaptable, widely distributed, drought tolerant and thrive in infertile soil, and can grow in a wide range of soil pH conditions, with high growth rates, high biomass yields, and low planting production costs (Anwar et al., 2007; He et al., 2019; Du et al., 2021a; Du et al., 2022c). In addition, the fresh branches and leaves of woody plants have a high crude protein (CP) content and are rich in various nutrients, such as bioactive components, amino acids, vitamins and macro minerals (Phesatcha and Wanapat, 2016; Oliveira et al., 2018; Wen et al., 2018; He et al., 2020; Du et al., 2021b).

Fresh branches and leaves of woody plants usually have a high moisture content, and the use of hay production methods not only increases the lignification of woody plant branches and leaves but also tends to cause leaf abscission during the drying process, resulting in a significant loss of nutrients (Du et al., 2021a). This suggests that hay processing is not suitable for the preparation of woody plant feeds. Silage, which is a fermented feed prepared from fresh forage for long-term storage, is considered a key technology for clean feed production of woody plant (Du et al., 2022a). In order to effectively utilize natural biomass resources, such as woody plant resources, to solve the problem of feed shortage and to improve the production capacity of livestock, this paper provides a comprehensive overview of the chemical composition and uses of feedable natural woody plants, the main factors affecting silage fermentation, the interactions between microbial co-occurrence networks and secondary metabolites, the regulatory mechanisms of silage fermentation, and the production of high-quality livestock products, with a view to providing important research information and technological support for the realization of the sustainable development of the animal husbandry.

## 2 Distribution and multifunctional utilization of woody plant

**Figure 1:**
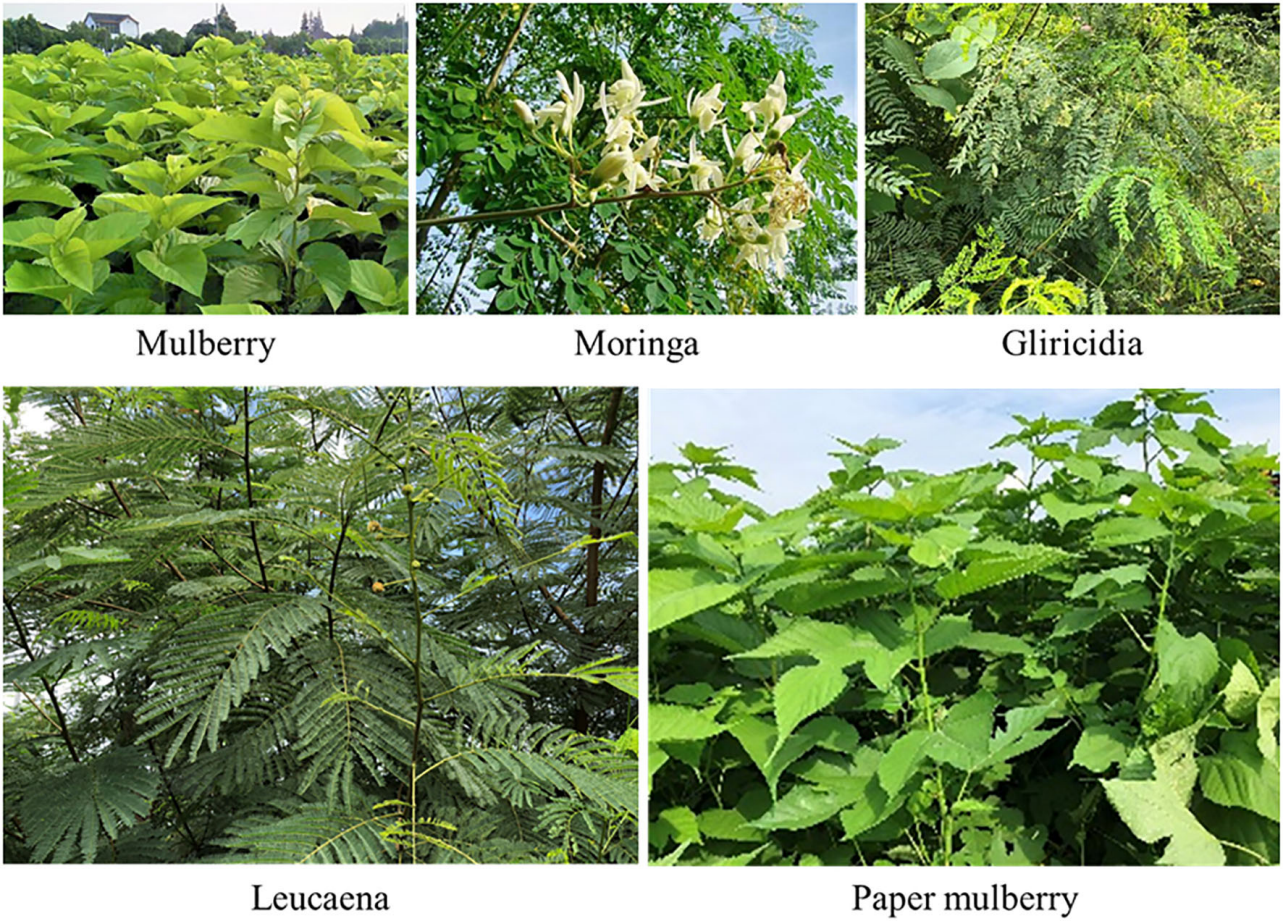
Woody plants that can be used as forage.

Forageable woody plants are characterized by their diversity and versatility, high biomass and rich nutrient content, making them suitable for feeding ruminants (**Figure 1**). Mulberry belongs to the family *Moraceae* and is native to north-central China. It is widely cultivated in China, Korea, Japan, Mongolia, India, Vietnam, Russia and other central Asian countries, as well as some European countries (Madhav and Carolyn, 2012). Moringa belongs to the family *Moraceae* and is found in the tropics of Africa and Asia, and is cultivated in Guizhou, Guangdong and Taiwan in China (Çelekli et al., 2019; Pagano et al., 2020). Gliricidia belongs to the family *Leguminosae* and is native to the tropical dry forests of Mexico and central America. In addition to its native range, it grows in many tropical and subtropical regions, including the Caribbean, northern parts of south America, central Africa, parts of India and southeast Asia, northern and central America, and central Africa (De Carvalho et al., 2017; Oliveira et al., 2018). Leucaena also belongs to the family *Leguminosae*, is native to southern Mexico and northern central America (Belize and Guatemala), and is cultivated in tropical regions (Rengsirikul et al., 2011). The paper mulberry belongs to the family *Moraceae* and is native to southwest China, but is now widely distributed throughout China, other Asian countries, mainland Europe and the Pacific islands (Peñailillo et al., 2016).

**Figure 2:**
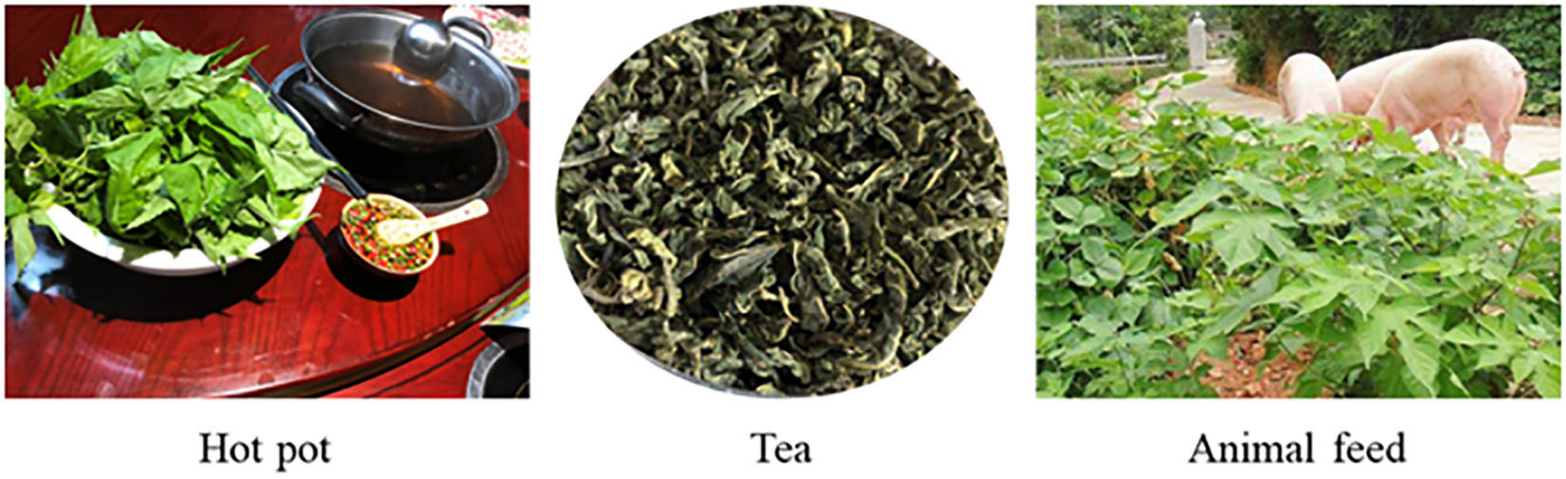
Multifunctional use of paper mulberry.

Woody plants have multiple uses (**Figure 2**), mainly as edible and medicinal products, but also as feed, compost, bioenergy and fiber products (Jitjaicham and Kusuktham, 2016; Wen et al., 2018; Hao et al., 2020; Ajayo et al., 2022). Because the fresh branches and leaves of woody plants are rich in nutrients and functional components, they can be used as a potential feed resource with additional value (Du et al., 2021b). In addition, woody plants provide economic benefits to farmers by reducing feed costs and increasing the productivity of livestock (Franzel et al., 2014). As shown in **Table 1**, all woody plants can be used as medicinal plants and as a source of feed for cattle, sheep, pigs and poultry. Some of them are used as raw materials for food, rabbit feed, bioenergy, biogas and green manure. In addition, paper mulberry and mulberry are used as a raw material for paper and fibre products, and mulberry leaves are an important feed for silkworm.

**Table 1:** Major use of woody plant.

Item	Feed use							Other use					
	Cow	Sheep	Goat	Pig	Poultry	Rabbit	Silkworm	Edible	Medicinal	Paper making	Bioenergy	Biogas and organic manure	Fabrics
Paper mulberry	○	○	○	○	○			○	○	○	○		○
Mulberry	○	○	○	○	○	○	○	○	○	○	○	○	○
Gliricidia	○	○	○	○	○	○			○		○	○	
Leucaena	○	○	○	○	○	○		○	○		○	○	
Moringa	○	○	○	○	○	○		○	○		○		

Data cited from the following references:

Paper mulberry from Singh et al., 1997; Cheng et al., 2001; Jitjaicham and Kusuktham, 2016; Hong et al., 2017; Si et al., 2018; Chen et al., 2020; Hao et al., 2020; Wang et al., 2020; Dong et al., 2021; Sheng et al., 2021; Tang et al., 2021; Xiong et al., 2021; Ajayo et al., 2022; Ma et al., 2022; Wu et al., 2022.

Mulberry from Janardhan et al., 2008; Kandylis et al., 2009; Dong et al., 2017; Kong et al., 2019; Amna et al., 2021; Ding et al., 2021; Liu et al., 2022; Maqsood et al., 2022; Martínez et al., 2005; Takasaki et al., 2011; Muck et al., 2018; Wang et al., 2018; Song et al., 2021; Tian et al., 2022.

Gliricidia from Nallathambi Gunaseelan, 1988; Mpairwe et al., 1998; Srinivasulu et al., 1999; Phimphachanhvongsod and Ledin, 2002; Kagya-Agyemang et al., 2007; Hariyadi and Hartono, 2018; Olugbenga et al., 2018; Isabel et al., 2019; Aulanni’Am et al., 2021; Marsetyo et al., 2021; Zhang et al., 2022.

Leucaena from Hussain et al., 1991; Maasdorp et al., 1999; Dana et al., 2000; Diaz et al., 2007; Giang et al., 2016; Harun et al., 2017; Chigurupati et al., 2020;

Soedarjo and Borthakur, 1996; Santos-Ricalde et al., 2017; Tudsri et al., 2019; Wang et al., 2021.

Moringa from Salem and Makkar, 2008; Mukumbo et al., 2014; Santos-Ricalde et al., 2017; Kholif et al., 2018; Fulvia et al., 2019; Hossam et al., 2019; Yang et al., 2020; Grosshagauer et al., 2021; Wu et al., 2021.

Some woody plants are rich in biofunctional components, such as polyphenols, carotenoids, alkaloids, terpenoids and sulphur-containing compounds, which have potent effects, including enhanced free radical scavenging and powerful reducing abilities. They also have anti-oxidant, anti-cancer, anti-inflammatory, hepatoprotective, hypotensive, anti-diabetic and hypolipidemic properties, and constitute therefore potential drugs for treating various diseases in humans and animals (Gopalakrishnan et al., 2016; Abd Rani et al., 2018). Gliricidia contains small amounts of coumarin, which can be used as a spice, but is generally not suitable for consumption (Lim, 2014). The leaves of woody plants, such as paper mulberry, mulberry, gliricidia and moringa, are rich in amino acids, fatty acids, vitamin E and beta-carotene, and their calcium and magnesium concentrations are higher than those of many vegetables. Therefore, they are used in many developing countries as leafy greens that provide plant-based protein and play a role in reducing hunger and combating malnutrition (Pakade et al., 2013). The leaves and seeds of woody plants can be eaten raw, cooked or added to food in the form of a dried powder. They are an ingredient in hot pots, teas, edible spices, beverages and yoghurt, and are therefore a popular health food in some Asian countries (Leone et al., 2015). The fruits of some woody plants contain large amounts of soluble sugars (Han et al., 2016), which are usually shed at maturity and decay on the ground, resulting in the loss of these resources. Using the fruits of these woody plants as a feedstock, biotechnology has been successfully developed to produce ethanol from their free sugars (Ajayo et al., 2022). The tree trunks of the paper mulberry and mulberry are rich in various chemical constituents, including cellulose, hemicellulose, lignin, waxes and gums, which are widely used in the production of paper and fibre products (Jitjaicham and Kusuktham, 2016). In addition, the leaves of mulberry, gliricidia and leucaena can be used as raw materials for biogas production and green leaf fertilizer (Tambone et al., 2020).

Woody plants are 90−99% organic matter (OM) and consist of 17−27% CP, 3−5% ether extract (EE), 11−21% true protein (TP) and 53−71% total digestible nutrient (TDN), with these ranges being generally higher than in forage crops and grasses (**Table 2**). The energy content and macro mineral (e.g. calcium, phosphorus, magnesium and potassium) concentrations in woody plant are also higher than in forage. Because woody plants are often utilized as fresh branches and leaves, their fibre and lignin contents are slightly different to those of forage. Woody plants contain the forage components required by livestock and are a high protein feed source. As a result, woody plants are referred to as “woody alfalfa” and their nutritional value is comparable to that of alfalfa (Zhang et al., 2019).

**Table 2:** Chemical composition, energy, and macro mineral of woody plant and forage.

Material	OM	CP	EE	NDF	ADF	ADL	NPN	TP	ADIP	TDN	NEl	NEm	NEg	Ca	P	Mg	K
	Chemical composition (% DM)						Protein composition (% DM)				Energy (Mcal/kg)			Macro mineral (g/kg DM)			
Woody plant																	
Paper mulberry	91.80	24.65	4.55	37.57	18.52	6.06	2.98	20.22	16.20	70.07	1.69	1.82	1.19	1.80	0.48	0.47	2.33
Mulberry	93.20	17.95	3.76	30.00	21.00	7.49	0.83	17.04	1.61	69.81	1.55	1.68	1.07	1.30	0.24	0.48	2.85
Gliricidia	90.30	25.91	4.02	52.10	34.52	11.09	3.26	18.36	8.94	56.71	1.21	1.29	0.72	1.57	0.27	0.59	2.64
Leucaena	92.62	26.31	3.41	60.62	37.49	13.40	4.14	18.59	12.03	53.19	1.11	1.18	0.62	1.26	0.23	0.42	2.56
Moringa	98.50	20.00	3.13	25.00	17.50	4.49	3.92	16.1	5.51	63.11	1.67	NF	NF	1.40	0.70	0.24	2.59
Forage																	
Corn	93.10	9.11	2.73	52.20	28.70	3.40	1.57	4.50	2.41	46.75	0.81	0.81	0.27	0.32	0.19	0.20	1.66
Sugarcane top	94.65	6.77	1.80	76.10	42.46	5.11	1.50	4.82	3.19	50.78	0.92	0.95	0.41	0.27	0.14	0.18	1.70
Alfalfa	81.30	15.93	4.80	47.30	39.70	7.60	1.48	3.50	10.59	51.70	1.36	1.34	0.72	1.41	0.26	0.26	2.60
Napier grass	85.72	5.56	1.35	66.74	41.53	5.68	1.62	3.43	2.10	41.18	0.64	0.61	0.08	0.41	0.41	0.50	2.06

OM, organic matter; CP, crude protein; EE, ether extract; NDF, neutral detergent fiber; ADF, acid detergent fiber; ADL, acid detergent lignin; DM, dry matter; NPN, non-protein nitrogen; TP, true protein; ADIP, acid detergent insoluble protein; TDN, total digestible nutrient; NEm, net energy for maintenance; NEl, net energy for lactation; NEg, net energy for gain; Ca, calcium; P, phosphorous; Mg, magnesium; K, potassium.

Data cited from the following references:

Paper mulberry from Du et al., 2022b; Mulberry from Zhang et al., 2020; Gliricidia and leucaena from Du et al., 2022c; Moringa from He et al., 2020; Corn from Wang et al., 2016; Sugarcane top from Cai et al., 2020a; Alfalfa from Liu et al., 2018; Napier grass from Cai et al., 2020b.

## 3 The main factor influencing fermentation feed of woody plant

Generally, forage crops and grasses grow well in the summer or tropical rainy season, but in the winter or dry season, cold and dry climatic conditions prevent forage crops from growing, resulting in the demand for feed from livestock far exceeding the production of feed (FAO, 2009). Therefore, woody plants have great potential for development as a feed resource for ruminant livestock, to make up for the shortage of feed. Woody plants are also harvested in large quantities in the summer and early autumn of temperate climates or in the tropical rainy season, and proper preparation and storage techniques after harvesting can effectively increase the self-sufficiency of local feed and the efficiency of livestock production (Su and Chen, 2020). Silage is a common traditional technique for the preparation and storage of forage crops and grasses and is an important means by which woody plants can be used effectively for feed production (Du et al., 2021b). The application of silage fermentation technology for the preparation of woody plant silage can avoid the loss of nutrients and enable long-term storage, thus solving the problem of livestock production during winter or drought seasons when feed is in short supply (Tao et al., 2020).

Silage is a stored feed made from fresh forage crops, grass and crop by-products, and is prepared by microbial fermentation under anaerobic conditions (Muck et al., 2018). Silage is widely used in many countries to make up for the lack of animal feed during the winter or in dry seasons. The fermentation of silage is influenced by various conditions, of which moisture, water-soluble carbohydrate (WSC), buffer energy and the epiphytic microbial community of the material are important factors affecting the fermentation process (McDonald et al., 1991). The appropriate moisture content for silage fermentation is 60−70%. Within this range lactic acid fermentation is promoted and the proliferation of spoilage microorganisms is inhibited. If the silage moisture content is too high, it tends to lead to butyric acid fermentation, dominated by clostridia, which decomposes proteins to produce ammonia nitrogen (NH_3_-N), thus reducing the fermentation quality of the silage. If the silage moisture content is too low, lactic acid bacteria (LAB) will be affected by the water activity and their growth will be inhibited, preventing them from producing large amounts of lactic acid to lower the pH of the silage. In addition, low moisture levels result in residual air not being effectively removed from the raw material, thus providing better conditions for harmful microorganisms such as aerobic bacteria and mold to survive, leading to poor quality fermentation (Du et al., 2022a). As shown in **Table 3**, the moisture content of fresh branches and leaves of woody plants can be as high as 70−80%, which is outside the ideal moisture range for silage fermentation. It is therefore necessary to make moisture adjustments when preparing woody plant silage. To make high quality silage, the material also needs to be above 6% WSC in dry matter (DM) and 10^5^ LAB colony-forming unit per gram (cfu/g) in fresh matter (FM). As shown in **Table 3**, both the WSC content and the LAB count of woody plants were below the theoretical threshold. Moreover, microorganisms harmful to silage fermentation, such as aerobic and coliform bacteria, were present at higher levels than the LAB count. These harmful microorganisms can compete with LAB for nutrients during ensiling and will affect the fermentation quality of silage. The lactic acid buffer capacity (LBC) is also an important factor affecting silage fermentation. Du et al. (2022c) reported that the LBC of woody plants is similar to that of leguminous grasses, generally above 700 mEq/kg of DM. The LBC strength of the silage material is closely related to the mineral composition, as shown in **Table 2** above. Woody plants are usually rich in mineral components such as K^+^, Ca^2+^ and Mg^2+^, and these cations neutralize the lactic acid and other organic acids produced by silage fermentation and inhibit the reduction of pH. This allows harmful microorganisms to grow and decompose proteins and produce NH_3_-N, resulting in low-quality silage fermentation (Cai et al., 2021). The chemical composition of woody plants in terms of moisture, protein, fiber and minerals varies at different growth stages and can directly affect the fermentation quality of silage (Du et al., 2021b; Du et al., 2022c). In addition, the growth stage and mixing ration of woody plant branches and leaves not only have an important influence on the fermentation quality of silage, but also on the digestibility and productivity of livestock (Anadón et al., 2014).

**Table 3:** Main factor affecting silage fermentation of woody plant.

Material	Moisture (%)	WSC (% DM)	LBC (mEq/kg DM)	Lactic acid bacteria	Harmful microbe
				Lg cfu/g FM	
Paper mulberry	81.25	4.14	892.27	4.66	5.43
Mulberry	71.06	4.92	637.3	4.11	5.68
Gliricidia	75.08	4.62	577.16	4.04	8.28
Leucaena	78.72	4.97	508.18	4.02	8.10
Moringa	80.20	4.88	506.71	3.56	3.87

WSC, water-soluble carbohydrate; LBC, lactic acid buffer capacity; DM, dry matter; cfu, colony-forming unit; FM, fresh matter. Harmful microbibe including aerobic bacteria, coliform bacteria, yeast and mold.

Data cited from the following references:

Paper mulberry from Du et al., 2022b; Mulberry from Zhang et al., 2020; Gliricidia and leucaena from Du et al., 2022c; Moringa from He et al., 2020.

## 4 Characterization of fermentation feed prepared with woody plant

Woody plants in cultivation or in the native state are usually harvested by harvesters or by hand and then used for silage preparation. In recent years, woody plants have been grown in greenhouses by tissue culture and mechanically harvested after cultivation at a growth height of about 1−1.5 m. They can be harvested 4−5 times a year in tropical or subtropical regions. The fresh branches and leaves of harvested woody plants can have a moisture content of up to 80%. When they are prepared and stored as hay the leaves will fall off, causing the nutrients to be lost during the drying and storage process. Furthermore, the drying process increases the lignin content and reduces the nutritional value and palatability of woody plants to livestock (Du et al., 2021b). Silage fermentation is therefore a good way of preserving the nutrients of woody plants and ensuring a year-round supply of feed for livestock through storage (Du et al., 2021b). Woody plant silage is prepared in the same way as forage crops and grasses, i.e. the woody plants are harvested at a suitable growth stage and the material is cut directly into 1−2 cm. To make good-quality silage, agricultural by-products such as rice bran, wheat bran and corn bran are usually added at 10-15% to regulate the moisture and then packed into silos or drums, sealed and covered for a period of time (Du et al., 2021c). Silage prepared in this way is usually well fermented and can store the nutrients of the silage for a long time.

**Figure 3:**
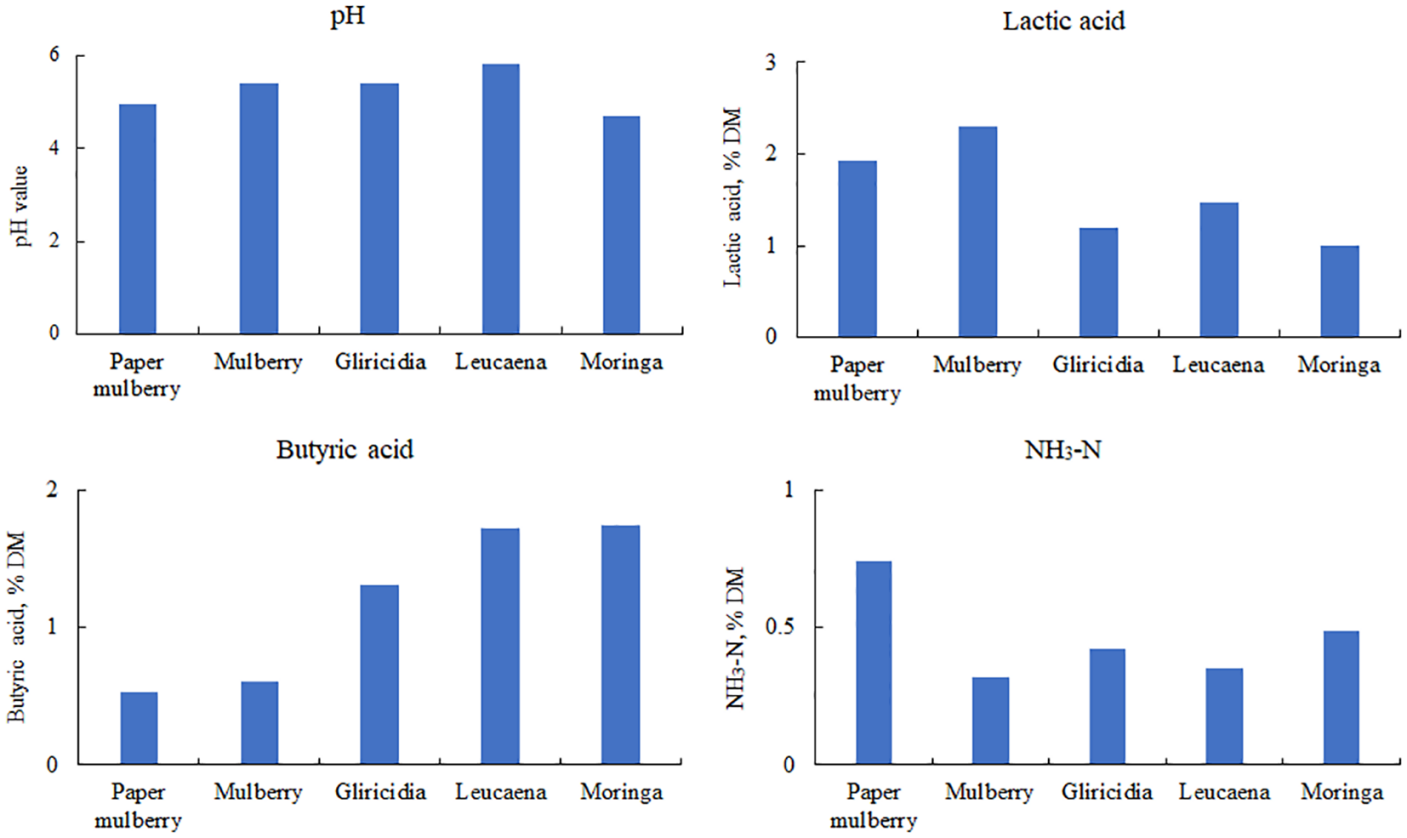
Fermentation characteristics of woody plant silage. DM, dry matter; NH_3_-N, ammonia nitrogen. Data cited from the following references: paper mulberry from Du et al., 2022b; Mulberry from Zhang et al., 2020; Gliricidia and leucaena from Du et al., 2022c; Moringa from He et al., 2020.

To investigate the natural fermentation characteristics of woody plant silage, fresh branches and leaves of woody plants were used as the raw material for silage preparation using a drumcan silo. Naturally fermented woody plant silage typically has a high pH, butyric acid and NH_3_-N content and a low lactic acid content (**Figure 3**). This is due to the high moisture content in the woody plant and the competitive use of WSC by the harmful epiphytic microorganisms, which prevents LAB from producing enough lactic acid to lower the pH during silage fermentation. This leads to butyric acid fermentation and the degradation of proteins to produce more NH_3_-N (Cai et al., 1999).

**Figure 4:**
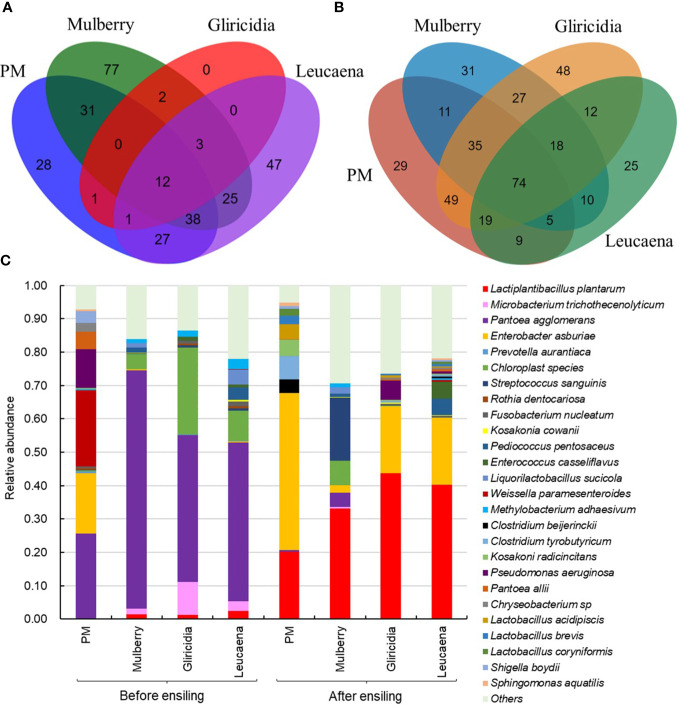
Microbial diversity and community structure of woody plant. **(A)** Venn diagram depicting unique or shared bacterial OTU of 97% sequence identity before ensiling; **(B)** Venn diagram after ensiling; **(C)** Relative abundances of the bacterial communities at the species levels. PM, paper mulberry. Data cited from the following references: Paper mulberry from Du et al., 2022b; Mulberry from Zhang et al., 2020; Gliricidia and leucaena from Du et al., 2022c.

To verify the natural fermentation characteristics of woody plant silage, a comprehensive analysis of the dynamic changes in microbial diversity and community structure during woody plant silage fermentation was conducted using the PacBio single molecule real-time (SMRT) sequencing technology. The Venn diagrams in **Figures 4A, B** clearly show the dynamics of the common and special microbial communities of woody plants before and after silage fermentation. Before ensiling, high moisture content woody plants under aerobic conditions are more suitable for aerobic microbial growth and, therefore, display high operational taxonomic unit (OTU) numbers and a rich microbial diversity. After ensiling, the double stress of the anaerobic and acidic environment created as fermentation progresses leads to the rapid death of some Gram-negative bacteria with thin cell walls. In the final stage of fermentation, Gram-positive bacteria that can adapt to an anaerobic and acidic environment, such as LAB, become the dominant microbial community and carry out lactic acid fermentation, lowering the pH and inhibiting the growth of harmful microorganisms, resulting in a significant decrease in the number and microbial diversity of OTUs (Méndez-García et al., 2015).

Among the microorganisms epiphytic to the fresh branches and leaves of woody plants, the relative abundance of LAB beneficial to silage fermentation is low, while the relative abundance of Gram-negative bacteria harmful to silage fermentation, such as *Pantoea*, *Enterobacter* and *Pseudomonas*, is high (**Figure 4C**). *Pantoea agglomerans*, the dominant bacterial community of woody plants, is a parthenogenic anaerobic Gram-negative pathogenic bacteria that is usually found on the surface of plants and is suitable for growth in neutral environments (Jacek et al., 2016). This kind of bacteria can compete with LAB for nutrients in the early stages of silage fermentation, breaking down glucose or other sugars and producing acids (Li and Nishino, 2013). *Enterobacter* constitute an aerobic or facultative anaerobic Gram-negative bacteria with a wide distribution and a large host range, which can be parasitic or symbiotic, epiphytic, saprophytic on humans, animals and plants, and can also survive in soil or water. If grown on plants, it can easily lead to blight (Si et al., 2023). *Pseudomonas* is a common aerobic or facultative anaerobic pathogen that prefers to live in moist environments, usually on soil, water, air and plants. It is designated as low pathogenic but is highly resistant to medication (Roberson and Firestone, 1992).

During ensiling, LAB can proliferate and use the WSC in the plant material to produce lactic acid and lower the pH. The anaerobic acidic environment that forms can play an important role in inhibiting the growth of harmful bacteria in the silage (Cai et al., 2020a). **Figure 4C** also confirms a significant increase in the relative abundance of LAB in woody plant silage, with *Lactiplantibacillus plantarum* becoming the main dominant species. This species of bacteria is able to respond rapidly to the dual stress of anaerobic and acidic ensiling environments, to carry out lactic acid fermentation and improve the fermentation quality of the silage. In addition, *Enterobacter* and *Citrobacter* have a proportional relative abundance in naturally fermented woody plant silage. *Citrobacter* constitute Gram-negative bacteria that utilize citrate as a sole source of carbon and may be associated with the production of citric acid aroma components in woody plant silage (Janda et al., 1994). This species of bacteria has a low acid tolerance and therefore has a low relative abundance in acidic silage environments. *Enterobacter* constitute some harmful silage bacteria that break down proteins in the early stages of silage fermentation, causing protein deamination and decarboxylation, which produce toxic compounds such as amines and branching fatty acids, resulting in foul-smelling silage (Ávila and Carvalho, 2020). This not only reduces the nutritional value of the silage and the palatability to the animal but can also have an impact on the hygiene and safety of the feed. Therefore, in terms of microbial community dynamics, naturally fermented woody plant silage does not reach a suitable level of quality and it is necessary to regulate the microbial community structure of the silage fermentation and promote lactic acid fermentation.

## 5 Regulating the anaerobic fermentation of woody plant

### 5.1 Fermentation regulation of woody plant silage by multiple preparation methods

To address the factors that hinder the fermentation of high-quality silage (i.e. high moisture content, strong LBC, low LAB count and low WSC content) in woody plants, rice bran and wheat bran have been added to adjust the moisture content and increase the fermentation substrate (Du et al., 2021a). Microbial additives, such as LAB, and cellulolytic enzymes have also been applied to improve the microbial community and increase the WSC content (Du et al., 2021b). In addition, hays including Napier grass and rice straw have been used to optimize the silage fermentation process of woody plants (Du et al., 2022a). The PacBio SMRT sequencing technology was applied to conduct an in-depth study of microbial diversity, co-occurrence microbial networks, metabolic pathways, final metabolites and the fermentation regulation mechanism of woody plant silage.

**Figure 5:**
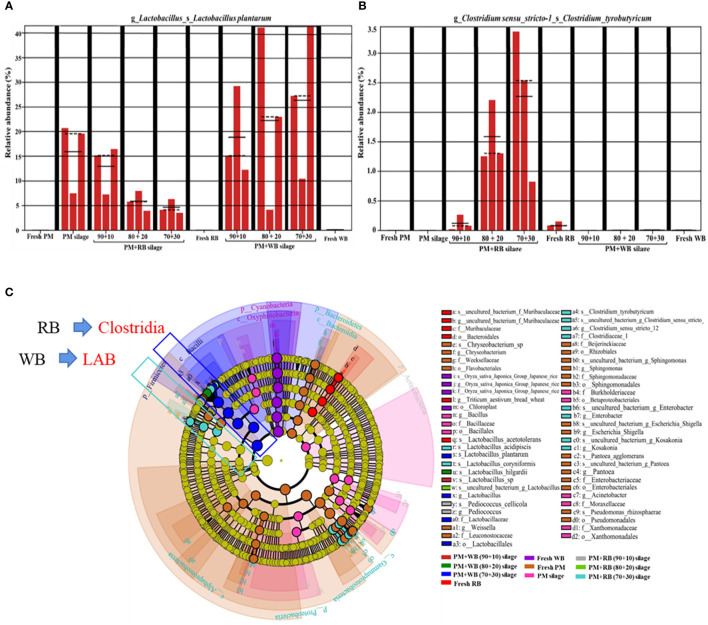
The community dynamics of lactic acid bacteria and clostridia in PM prepared with RB and WB before and after ensiling. **(A)** metaStat analysis of *Lactobacillus plantarum*; **(B)** metaStat analysis of *Clostridium tyrobutyricum*; **(C)** cladogram comparison of bacterial community. PM, paper mulberry; RB, rice bran; WB, wheat bran. The figure cited from Du et al., 2021a.

The moisture content of the rice bran and wheat bran used in the study was less than 10%. When mixed with woody plants at a rate of 10−30% of the FM, the moisture was adjusted to exactly 60−70% (Du et al., 2021a), which is the ideal moisture range for silage preparation. Woody plants have a similar epiphytic microbial community structure to forage crops and grasses, i.e. they have a higher relative abundance of harmful microorganisms and a lower relative abundance of LAB in the fresh material. The relative abundance of epiphytic *L. plantarum* from the woody plant material was below the detection level, but increased significantly after ensiling (**Figure 5A**). Thus, the aerobic environment created by woody plants prior to ensiling was not beneficial for the competitive growth of LAB, while aerobic microorganisms were at an advantage. **Figure 5B** shows that the relative abundance of clostridia in the mixed silage of woody plants and wheat bran was below the detection level, but the mixed silage of woody plants with rice bran increased the relative abundance of clostridia. Wheat bran effectively regulated the moisture and increased the fermentation substrate of woody plants, and the anaerobic conditions created by silage fermentation accelerated the succession of LAB, which became the dominant community and inhibited the proliferation of clostridia. The silage prepared with 30% wheat bran and 70% woody plants had the best microbial community structure. The addition of rice bran also served to adjust the moisture and fermentation substrate, but rice bran can sometimes be enriched with *Clostridium* spp., which is a strictly anaerobic Gram-positive bacteria whose spores tolerate the acidic and anaerobic environmental conditions of silage (Li et al., 2020). Clostridia breaks down sugars, organic acids and proteins during the silage process, producing butyric acid, NH_3_-N, carbon dioxide (CO_2_) and hydrogen (H_2_), thereby reducing the fermentation quality of the silage (Cassir et al., 2016). In addition, some proportion of clostridia is pathogenic and can be harmful to the health of livestock (Uzal et al., 2018). The microbial community dynamics in **Figure 5C** also confirmed that *L. plantarum* and *Clostridium typhimurium* were the two dominant bacteria in woody plant silage mixed with rice bran and wheat bran. There were significant differences in the relative abundance of these two species compared to other silage bacterial communities. Therefore, compared to rice bran, wheat bran is a more suitable exogenous addition to the silage fermentation of woody plants, not only to regulate the fermentation conditions but also to improve the microbial community structure, thus promoting woody plant silage fermentation.

In addition, the fermentation characteristics of woody plant silage were explored by applying additions such as LAB inoculant and cellulolytic enzymes (Du et al., 2021b). Microbial additives can cause a significant decrease in microbial diversity in woody plant silage and the microbial community rapidly completes a dynamic succession process from Gram-negative to Gram-positive bacteria. In the anaerobic and acidic environment created by silage fermentation, Gram-negative bacteria such as *Enterobacter asburiae* die off rapidly, while LAB respond rapidly to the anaerobic conditions of silage and become the dominant community, dominating the fermentation process. Thus, microbial additives influence silage fermentation by improving the microbial community. In addition, the use of locally available low-cost crop straw and hay mixed with woody plants for silage making is a viable option for improving the silage fermentation quality. The results showed that the addition of Napier grass and rice straw allowed *L. plantarum* and *Lactococcus cellulosus* to act synergistically with each other, enabling LAB rapidly to become the dominant community for woody plant silage, and that the addition of 10−30% hay was effective in improving the fermentation quality of woody plant silage.

### 5.2 Mechanism of interaction between co-occurrence microbial networks and secondary metabolites during silage fermentation

The metabolites produced by silage microorganisms during fermentation have a strong influence on the fermentation quality, flavor and aerobic spoilage of silage (Ávila and Carvalho, 2020). Silage fermentation forms a microbial co-occurrence network system, which includes a complex process of dynamic succession of the microbial community and changes in their metabolites, which vary greatly from the different microorganisms through their metabolic pathways (Du et al., 2022c). The LAB produce metabolites, such as organic acids, ethanol, 1,2 propylene glycol and biogenic amines during silage fermentation, some of which play an important role in inhibiting the growth of harmful bacteria and improving aerobic stability (Guo et al., 2018). The LAB usually use WSC to produce lactic acid and lower the pH, which can improve the fermentation quality of silage (Okoye et al., 2022). In contrast, *Enterobater* species can ferment glucose to produce succinic acid, lactic acid, acetic acid, formic acid and ethanol, as well as producing CO_2_ and H_2_ gas, and increasing the DM and energy loss (Thompson et al., 2008). In addition, some metabolites such as lactic and acetic acid reduce the pH and inhibit the growth of aerobic bacteria and molds, which can improve the fermentation quality and aerobic stability of silage (Cai et al., 2020a). Thus, the microbial community structure and metabolites interact and influence the fermentation quality of silage.

**Figure 6:**
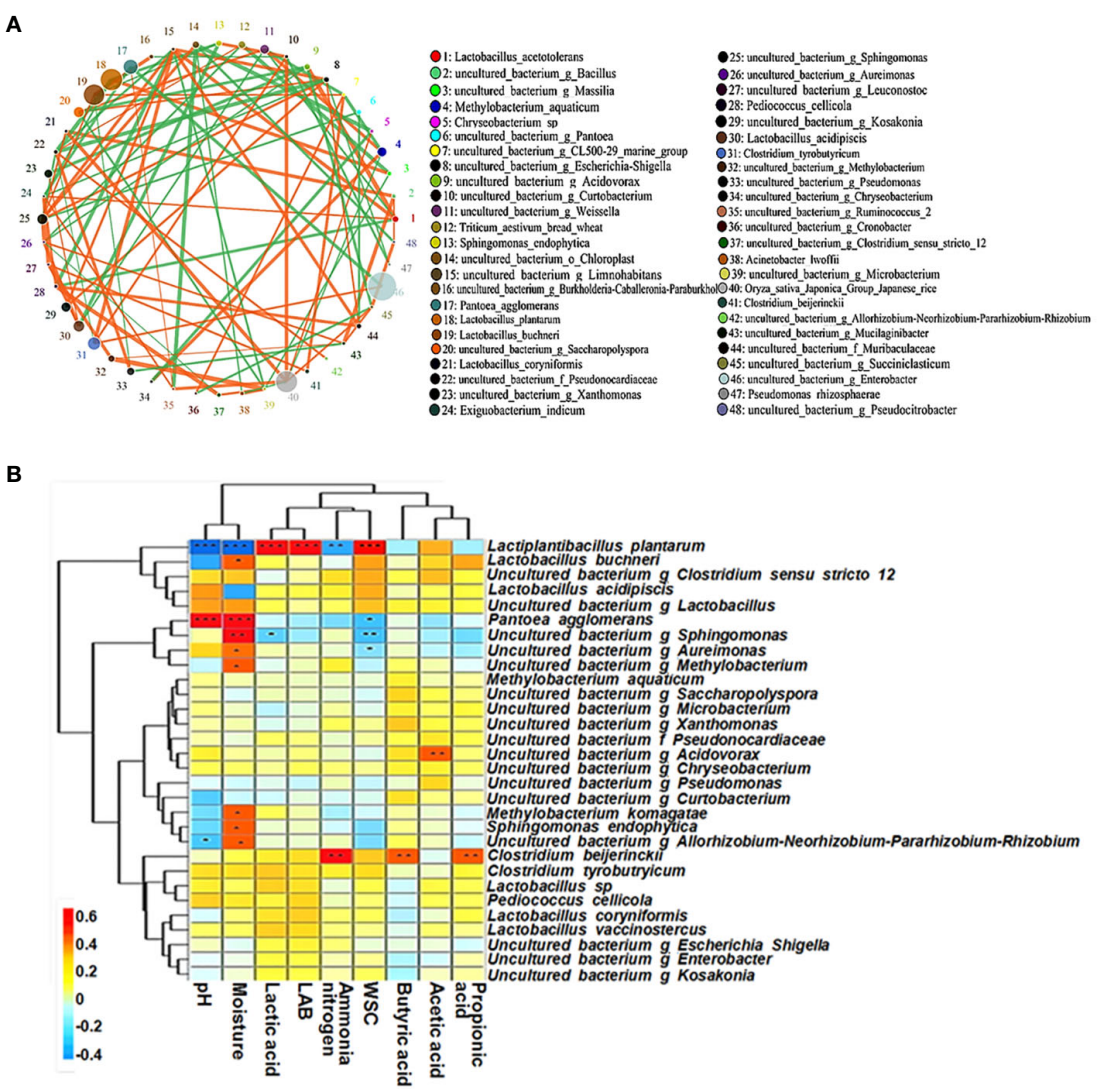
The microbial co-occurrence network **(A)** and correlation analyses between bacterial community and terminal fermentation product **(B)** at species level in paper mulberry silage. The figure cited from Du et al., 2021a.

The abundance levels of harmful microorganisms such as *Enterobacter* spp. and *Clostridium tyrobutyricum* were positively correlated, which may be related to the low-quality fermentation of the silage (**Figure 6A**). As silage fermentation progressed, *L. plantarum* rapidly formed the dominant community, which in turn replaced the dominant population of the harmful bacteria *P. agglomerans* in the early stages of fermentation.

A positive correlation was found between the moisture content and *P. agglomerans*, *Sphingomonas* spp., *Aureimonas* spp. and *Methylobacterium* spp. and between WSC and LAB (**Figure 6B**). This suggests that a high moisture content silage environment encourages the proliferation of these harmful bacteria and that WSC can promote the growth of LAB. In addition, LAB produce lactic acid during ensiling, which inhibits the growth of these harmful bacteria, thereby lowering pH and reducing the production of NH_3_-N. *Clostridium beijerinckii* form a nutrient-competitive interrelationship with LAB, i.e. *C. beijerinckii* undergo butyric acid fermentation, which promotes the production of propionic and butyric acids and hinders the proliferation of LAB. *Acidovorax* spp. are aerobic or facultative anaerobic Gram-negative bacteria that can use residual oxygen to produce acetic acid in the early stages of silage fermentation (Du et al., 2021a). Therefore, Gram-positive and Gram-negative bacteria (e.g. *L. plantarum*) form a mutually constraining opposition during silage fermentation; their community structure and metabolites interact with each other and influence the fermentation quality of the silage.

**Figure 7:**
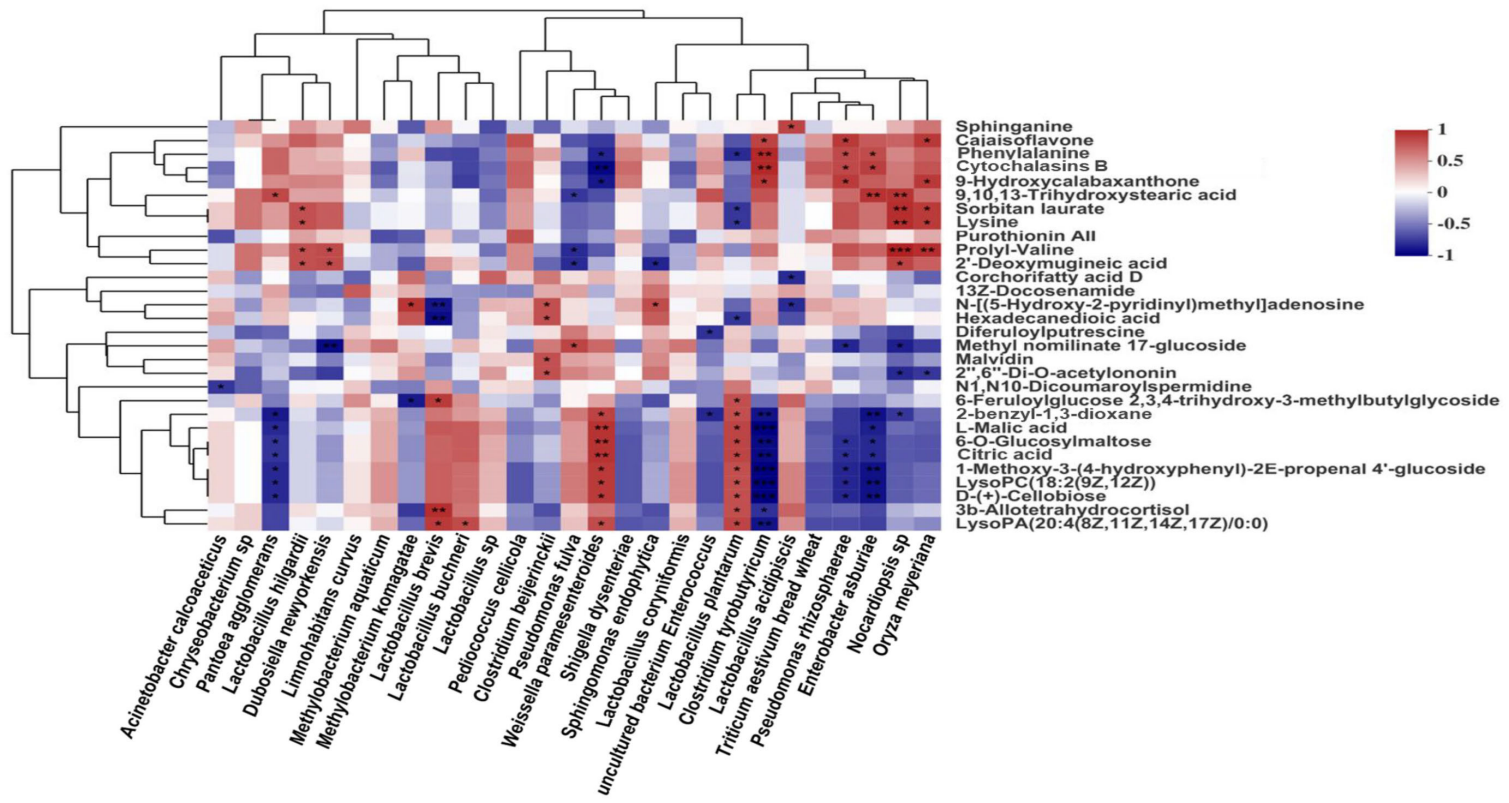
Correlations between microbial community and metabolites in silage. Microbial community, main bacterial species; metabolites, differentially presented metabolites. The figure cited from Du et al., 2022b.

To explore the fermentation regulation mechanism of woody plant silage, non-targeted metabolomics techniques were used to study the mechanisms of interaction between the co-occurrence microbial networks and secondary metabolites of woody plant silage prepared with bran (Du et al., 2022a). A Spearman correlation analysis of the main bacterial community and metabolites of silage showed that the aromatic compound-like metabolites of silage were positively correlated with LAB, such as *L. plantarum* and *Weissella paramesenteroides*, and negatively correlated with other bacterial community members, such as *C. tyrobutyricum* and *E. asburiae* (**Figure 7**). Among the aromatic compounds, citric acid and L-malic acid are key intermediates in the tricarboxylic acid cycle metabolic pathway, which is produced by most LAB. Citric acid is formed by the carboxylation of acetyl coenzyme A and oxaloacetate in the tricarboxylic acid cycle and is involved in the metabolism of sugars, fats and proteins (Ke et al., 2017). During silage fermentation, citric acid can play a role in lowering silage pH and inhibiting the activity of undesirable fermentation fungi, such as yeasts and moulds. In addition, LAB in the silage fermentation process can metabolize citric acid to produce diacetyl and acetic acids, and other substances with flavor, which can improve the flavor of silage and the palatability to livestock. When animals are fed silage containing citric acid the proliferation of pathogens can be reduced and the production of toxic metabolites can be inhibited, which can improve the stress capacity of livestock. L-malic acid is an important natural organic acid with antioxidant properties that regulates silage pH and promotes the growth of LAB (Wang et al., 2009). During silage fermentation, LAB with the function of saccharifying starch can use starch directly for fermentation, thus producing L-malic acid, which will play a role in improving the silage fermentation quality. In addition, LAB can use D-(+)-cellobiose as a substrate for energy production, accelerating growth and inhibiting the proliferation of yeast, mold, clostridia and enterobacter. These findings suggest that silage microbiomics are closely linked to metabolomics. The microbial community structure can influence the types of final and secondary metabolites, which in turn are important factors influencing the fermentation quality of silage.

## 6 Woody plant and livestock product

To popularize the use of woody feed resources in livestock production, feeding experiments have been conducted using a variety of livestock, including cattle, sheep and poultry.

In experiments with fed cattle, Holstein cows fed total mixed fermentation (TMR) formulated with 10−15% paper mulberry instead of maize silage, alfalfa hay and oat hay had a similar DM intake and milk production to those fed with conventional TMR. However, the addition of paper mulberry to TMR diets significantly increased the serum levels of immunoglobulin A (IgA), immunoglobulin (IgG), catalase and superoxide dismutase and the total antioxidant capacity, but decreased the levels of 8-hydroxy-2′-deoxyguanosine. In addition, paper mulberry TMR can cause a significant decrease in the milk somatic cell count, enhance the immune and antioxidant function of cows and increase the polyunsaturated fatty acid content of milk (Si et al., 2018). The fresh branches and leaves of mulberry are rich in flavonoids and feeding them to weaned Holstein calves promotes the average daily gain and feed efficiency (Kong et al., 2019). Gliricidia leaves and elephant ear pods are rich in tannins and saponins. When a mixture of the two was fed to beef cattle, it increased the digestible CP intake, inhibited methanogenic bacteria in the rumen and reduced methane emissions (Isabel et al., 2019). Leucaena silage improves the DM intake, feed digestibility and rumen end fermentation products in dairy cows and reduces methane production (Giang et al., 2016). A TMR including moringa improves the milk fat content and levels of secondary metabolites, regulates rumen fermentation and inhibits methane production by dairy cattle (Dong et al., 2019).

In sheep feeding trials, replacing some of the soybean meal and corn stover with paper mulberry silage reduced total volatile fatty acids in the rumen, and increased DM intake and average daily gain of lambs (Xiong et al., 2021). When some of the hay and concentrate in the TMR was replaced by mulberry leaves, there was no significant difference in the digestibility of DM, CP, and crude fibre in sheep compared to the conventional TMR (Kandylis et al., 2009). In addition, silage prepared from a mixture of gliricidia with cassava improves growth performance, digestibility, feeding behavior and the carcass characteristics of lambs, and increases the yield of key commercial meats, such as loin and ham, as well as typical foodstuffs, such as lamb (Oliveira et al., 2018). Feed supplementation with moringaleaf extract increased milk production, reduced saturated fatty acids and increased the levels of unsaturated fatty acids and conjugated linoleic acid in goats (Kholif et al., 2018).

In poultry feeding trials, the use of woody plants, such as paper mulberry and mulberry, in place of some of the commercial feeds maintained good indicators in terms of the egg production rate, egg weight, fertilized egg hatching rate, growth performance and meat quality (Yang et al., 2020). There is great future potential for seedling production and breeding of woody plants for healthy livestock feeding with antibiotic substitutes.

## 7 Conclusion

**Figure 8:**
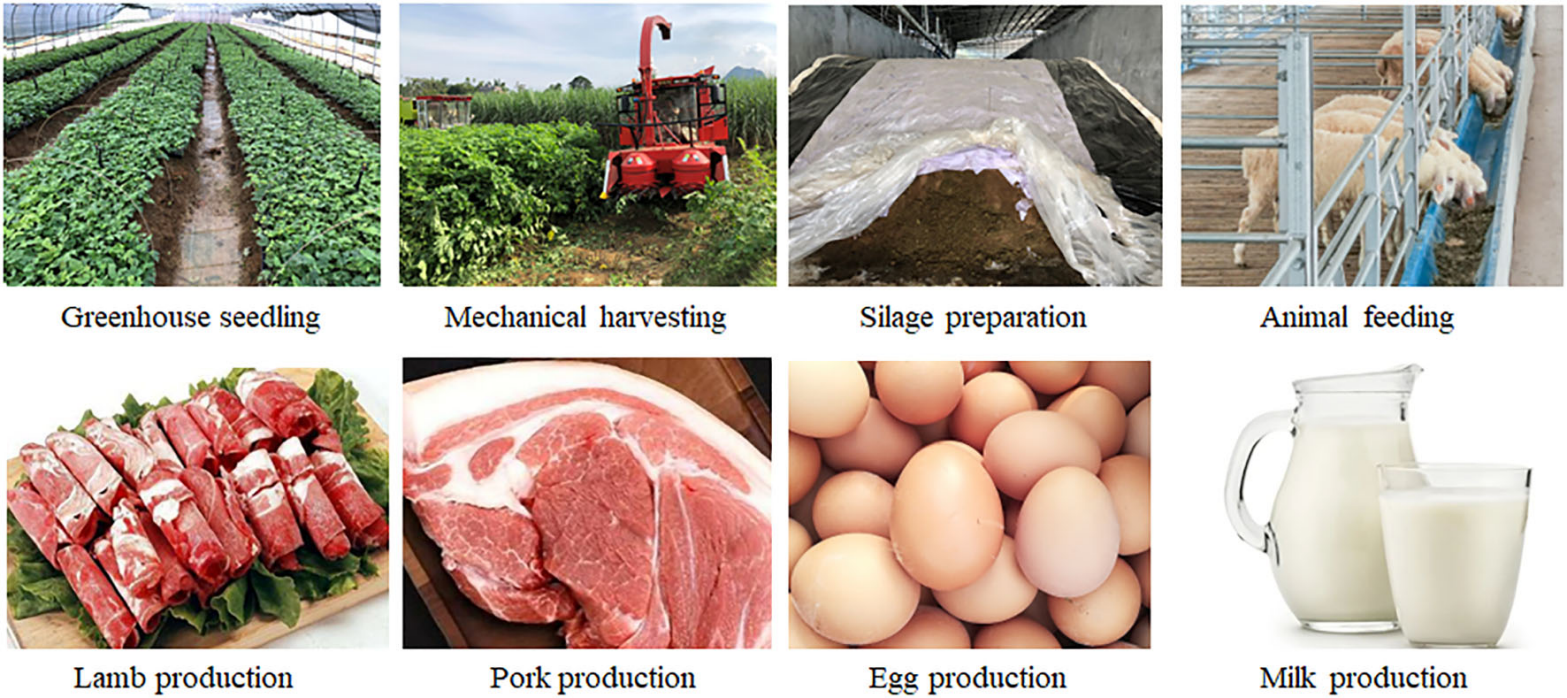
Production potential of feed and livestock product from woody plant.

To develop and utilize new woody feed resources to produce high-quality livestock products, this review provided a comprehensive overview of the composition and uses of natural woody plants that can be fed to animals, the dominant factors affecting woody plant silage fermentation, microbial succession patterns and the mechanisms of interaction between microbial co-occurrence networks and secondary metabolites. Woody plants are rich in nutrients and can be used for the preparation of fermented feeds and the production of value-added livestock products (**Figure 8**). This has important implications for alleviating feed shortages and promoting sustainable development of animal husbandry.

## Author contributions

ZD and YC: Writing - original draft, Investigation. ZD, YC, and FY: Writing–original draft, Formal analysis. ZD and JF: Validation, Writing−review & editing; SY: Conceptualization, Visualization. TO, DN, and HK: Supervision, Resources. YC and FY: Resources, Writing−review & editing, Supervision, Funding acquisition. All authors contributed to the article and approved the submitted version.

## References

[B1] Abd Rani N. Z. Husain K. Kumolosasi E. (2018). Moringa genus: A review of phytochemistry and pharmacology. Front. Pharmacol. 9. doi: 10.3389/fphar.2018.00108 PMC5820334 29503616

[B2] Ajayo P. C. Huang M. Zhao L. Tian D. Jiang Q. Deng S.. (2022). Paper mulberry fruit juice: A novel biomass resource for bioethanol production. Bioresour. Bioprocess. 9, 3. doi: 10.1186/s40643-021-00490-3 PMC10991237 38647748

[B3] Amna T. Hassan M. S. Sheikh F. A. Seo H. C. Kim H. C. Alotaibi N.. (2021). Natural mulberry biomass fibers doped with silver as an antimicrobial textile: A new generation fabric. Text. Res. J. 91 (21-22), 2581–2587. doi: 10.1177/004051752110134

[B4] Anadón J. D. Sala O. E. Turner B. L. Bennett E. M. (2014). Effect of woody-plant encroachment on livestock production in North and South America. PNAS 111 (35), 12948–12953. doi: 10.1073/pnas.1320585111 25136084 PMC4156688

[B5] Anwar F. Latif S. Ashraf M. Gilani A. H. (2007). Moringa oleifera: A food plant with multiple medicinal uses. Phytother. Res. 21 (1), 17–25. doi: 10.1002/ptr.2023 17089328

[B6] Aulanni’Am A. Ora K. M. Ariandini N. A. Wuragil D. K. Permata F. S. Riawan W.. (2021). Wound healing properties of Gliricidia sepium leaves from Indonesia and the Philippines in rats (Rattus norvegicus). Vet. World 14 (3), 820–824. doi: 10.14202/vetworld.2021.820-824 33935433 PMC8076467

[B7] Ávila C. L. S. Carvalho B. F. (2020). Silage fermentation-updates focusing on the performance of micro-organisms. J. Appl. Microbiol. 128 (4), 966–984. doi: 10.1111/jam.14450 31519038

[B8] Cai Y. Benno Y. Ogawa M. Kumai S. (1999). Effect of applying lactic acid bacteria isolated from forage crops on fermentation characteristics and aerobic deterioration of silage. J. Dairy. Sci. 82 (3), 520–526. doi: 10.3168/jds.S0022-0302(99)75263-X 10194670

[B9] Cai Y. Du Z. Jethro D. B. Nignan M. Yamasaki S. (2021). Analysis of main factors affecting silage fermentation of sorghum prepared with whole crop and stover in semiarid West Africa. Afr. J. Range For. Sci. 38 (2), 169–178. doi: 10.2989/10220119.2020.1794959

[B10] Cai Y. Du Z. Yamasaki S. Nguluve D. Tinga B. Macome F.. (2020a). Community of natural lactic acid bacteria and silage fermentation of corn stover and sugarcane tops in Africa. Asian-Austral. J. Anim. Sci. 33 (8), 1252–1264. doi: 10.5713/ajas.19.0348 PMC7322639 32054211

[B11] Cai Y. Du Z. Yamasaki S. Nguluve D. Tinga B. Macome F.. (2020b). Influence of microbial additive on microbial populations, ensiling characteristics, and spoilage loss of delayed sealing silage of Napier grass. Asian-Austral. J. Anim. 33, 1103–1112. doi: 10.5713/ajas.19.0471 PMC7322651 31480132

[B12] Cassir N. Benamar S. La Scola B. (2016). Clostridium butyricum: From beneficial to a new emerging pathogen. Clin. Microbiol. Infect. 22 (1), 37–45. doi: 10.1016/j.cmi.2015.10.014 26493849

[B13] Çelekli A. Al-Nuaimi A. I. Bozkurt H. (2019). Adsorption kinetic and isotherms of Reactive Red 120 on Moringa oleifera seed as an eco-friendly process. J. Mol. Struct. 1195, 168–178. doi: 10.1016/j.molstruc.2019.05.106

[B14] Chen G. Shui S. Chai M. Wang D. Su Y. Wu H.. (2020). Effects of paper mulberry (Broussonetia papyrifera) leaf extract on growth performance and fecal microflora of weaned piglets. BioMed. Res. Int. 2020, 6508494. doi: 10.1155/2020/6508494 33274217 PMC7700021

[B15] Cheng Z. Lin C. Hwang T. Teng C. (2001). Broussochalcone a, a potent antioxidant and effective suppressor of inducible nitric oxide synthase in lipopolysaccharide-activated macrophages. Biochem. Pharmacol. 61 (8), 939–946. doi: 10.1016/S0006-2952(01)00543-3 11286985

[B16] Chigurupati S. Vijayabalan S. Selvarajan K. K. Alhowail A. Kauser F. (2020). Bacterial endosymbiont inhabiting Leucaena leucocephala leaves and their antioxidant and antidiabetic potential. J. Complement. Integr. Med. 18 (2), 319–325. doi: 10.1515/jcim-2020-0203 34187119

[B17] Dana N. Tegegne A. Shenkoru T. (2000). Feed intake, sperm output and seminal characteristics of Ethiopian highland sheep supplemented with different levels of leucaena (Leucaena leucocephala) leaf hay. Anim. Feed. Sci. Tech. 86 (3-4), 239–249. doi: 10.1016/S0377-8401(00)00152-8

[B18] De Carvalho G. G. P. Rebouças R. A. Campos F. S. Santos E. M. Araújo G. G. L. Gois G. C.. (2017). Intake, digestibility, performance, and feeding behavior of lambs fed diets containing silages of different tropical forage species. Anim. Feed. Sci. Tech. 228, 140–148. doi: 10.1016/j.anifeedsci.2017.04.006

[B19] Diaz C. Grageola F. Lemus C. Ly J. (2007). Studies on the cell wall digestibility in pigs fed leucaena (Leucaena leucophala (Lam.) de Wit) leaf meal. J. Anim. Vet. Adv. 6 (10), 1190–1193. doi: 10.2460/javma.231.7.1098

[B20] Ding Y. Jiang X. Yao X. Zhang H. Song Z. He X.. (2021). Effects of feeding fermented mulberry leaf powder on growth performance, slaughter performance, and meat quality in chicken broilers. Animals 11 (11), 3294. doi: 10.3390/ani11113294 34828025 PMC8614317

[B21] Dong Z. Ding Z. Zhang S. Zhang Y. Fan H. Yang Y. (2017). Natural fibers from the bark of mulberry branches for textile application. Fibers. Text. East. Eur. 25 (3), 20–25. doi: 10.5604/10.5604/01.3001.0010.1684

[B22] Dong Y. Li P. Li P. Chen C. (2021). First comprehensive analysis of lysine succinylation in paper mulberry (Broussonetia papyrifera). BMC Genomics 22 (1), 255. doi: 10.1186/s12864-021-07567-5 33838656 PMC8035759

[B23] Dong L. Zhang T. Diao Q. (2019). Effect of dietary supplementation of moringa oleifera on the production performance and fecal methanogenic community of lactating dairy cows. Animals 9 (5), 262. doi: 10.3390/ani9050262 31121857 PMC6562924

[B24] Du Z. Lin Y. Sun L. Yang F. Cai Y. (2021a). Microbial community structure, co-occurrence network and fermentation characteristics of woody plant silage. J. Sci. Food Agr. 102 (3), 1193–1204. doi: 10.1002/jsfa.11457 34343355

[B25] Du Z. Sun L. Chen C. Lin J. Yang F. Cai Y. (2021b). Exploring microbial community structure and metabolic gene clusters during silage fermentation of paper mulberry, a high-protein woody plant. Anim. Feed. Sci. Tech. 275, 114766. doi: 10.1016/j.anifeedsci.2020.114766

[B26] Du Z. Sun L. Lin Y. Chen C. Yang F. Cai Y. (2022a). Use of Napier grass and rice straw hay as exogenous additive improves microbial community and fermentation quality of paper mulberry silage. Anim. Feed. Sci. Tech. 285, 115219. doi: 10.1016/j.anifeedsci.2022.115219

[B27] Du Z. Sun L. Lin Y. Yang F. Cai Y. (2021c). The use of PacBio SMRT technology to explore the microbial network and fermentation characteristics of woody silage prepared with exogenous carbohydrate additives. J. Appl. Microbiol. 131 (5), 2193–2211. doi: 10.1111/jam.15124 33905586

[B28] Du Z. Sun L. Lin Y. Yang F. Cai Y. (2022b). Using PacBio SMRT sequencing technology and metabolomics to explore the microbiota-metabolome interaction related to silage fermentation of woody plant. Front. Microbiol. 13. doi: 10.3389/fmicb.2022.857431 PMC9251423 35794909

[B29] Du Z. Yamasaki S. Oya T. Nguluve D. Euridse D. Tinga B.. (2022c). Microbial co-occurrence network and fermentation information of natural woody-plant silage prepared with grass and crop by-product in Southern Africa. Front. Microbiol. 13. doi: 10.3389/fmicb.2022.756209 PMC8964296 35369476

[B30] FAO. (2009). “The state of food and agriculture,” in Livestock in the balance (Rome, Italy: Food and Agriculture Organization of the United Nations).

[B31] Franzel S. Carsan S. Lukuyu B. Sinja J. Wambugu C. (2014). Fodder trees for improving livestock productivity and smallholder livelihoods in Africa. Curr. Opin. Env. Sust. 6, 98–103. doi: 10.1016/j.cosust.2013.11.008

[B32] Fulvia T. Matteo P. Floriana B. Fabrizio A. (2019). Moringa oleifera Lam. As an energy crop for biogas production in developing countries. Biomass Convers. Bior. 10, 1083–1089. doi: 10.1007/s13399-019-00550-x

[B33] Giang N. T. T. Wanapat M. Phesatcha K. Kang S. (2016). Level of Leucaena leucocephala silage feeding on intake, rumen fermentation, and nutrient digestibility in dairy steers. Trop. Anim. Health Pro. 48, 1057–1064. doi: 10.1007/s11250-016-1060-3 27113453

[B34] Gopalakrishnan L. Doriya K. Kumar D. S. (2016). Moringa oleifera: A review on nutritive importance and its medicinal application. Food Sci. Hum. Well. 5 (2), 49–56. doi: 10.1016/j.fshw.2016.04.001

[B35] Grosshagauer S. Pirkwieser P. Kraemer K. Somoza V. (2021). The future of moringa foods: A food chemistry perspective. Front. Nutr. 8. doi: 10.3389/fnut.2021.751076 PMC8594418 34796194

[B36] Guo X. S. Ke W. C. Ding W. R. Ding L. M. Xu D. M. Wang W. W.. (2018). Profiling of metabolome and bacterial community dynamics in ensiled Medicago sativa inoculated without or with Lactobacillus plantarum or Lactobacillus buchneri. Sci. Rep-UK. 8 (1), 357. doi: 10.1038/s41598-017-18348-0 PMC5762819 29321642

[B37] Han Q. Wu Z. Huang B. Sun L. Ding C. Yuan S.. (2016). Extraction, antioxidant and antibacterial activities of Broussonetia papyrifera fruits polysaccharides. Int. J. Biol. Macromol. 92, 116–124. doi: 10.1016/j.ijbiomac.2016.06.087 27370746

[B38] Hao Y. Huang S. Si J. Zhang J. Gaowa N. Sun X.. (2020). Effects of paper mulberry silage on the milk production, apparent digestibility, antioxidant capacity, and fecal bacteria composition in Holstein dairy cows. Animals 10, 1152. doi: 10.3390/ani10071152 32645955 PMC7401539

[B39] Hariyadi F. H. P. Hartono A. (2018). Biomass and carbon stock potential of Gliricidia Sepium as an alternative energy at Timor Tengah Utara Regency, East Nusa Tenggara Province, Indonesia. IOP. Conf. Series.: Earth Env. Sci. 141 (1), 12022. doi: 10.1088/1755-1315/141/1/012022

[B40] Harun N. L. A. Alimon A. R. Jahromi M. F. Samsudin A. A. (2017). Effects of feeding goats with Leucaena leucocephala and Manihot esculenta leaves supplemented diets on rumen fermentation profiles, urinary purine derivatives and rumen microbial population. J. Appl. Anim. Res. 45 (1), 409–416. doi: 10.1080/09712119.2016.1205499

[B41] He L. Zhou W. Wang C. Yang F. Chen X. Zhang Q. (2019). Effect of cellulase and Lactobacillus casei on ensiling characteristics, chemical composition, antioxidant activity, and digestibility of mulberry leaf silage. J. Dairy. Sci. 102 (11), 9919–9931. doi: 10.3168/jds.2019-16468 31447142

[B42] He L. Zhou W. Xing Y. Pian R. Chen X. Zhang Q. (2020). Improving the quality of rice straw silage with Moringa oleifera leaves and propionic acid: Fermentation, nutrition, aerobic stability and microbial communities. Bioresour. Technol. 299, 122579. doi: 10.1016/j.biortech.2019.122579 31855660

[B43] Hong H. Jo H. J. Kim S. J. (2017). The physical properties of handmade Jumchi-Hanji made with Korea paper mulberry. J. Korean. Soc. Clothing. Textiles. 41 (4), 633–645. doi: 10.5850/JKSCT.2017.41.4.633

[B44] Hossam M. E. Ahmed E. K. Maria C. Uchenna Y. A. (2019). Ruminal fermentation kinetics of Moringa oleifera leaf and seed as protein feeds in dairy cow diets: In sacco degradability and protein and fiber fractions assessed by the CNCPS method. Agroforest. Syst. 94, s905–s915. doi: 10.1007/s10457-019-00456-7

[B45] Hussain J. Reddy P. V. V. S. Reddy V. R. (1991). Utilisation of leucaena leaf meal by broilers. Brit. Poultry. Sci. 32 (1), 131–137. doi: 10.1080/00071669108417334

[B46] Isabel C. M. Julian A. Sara V. Rolando B. Carlos F. A. Armín A. B.. (2019). Effects of tannins and saponins contained in foliage of Gliricidia sepium and pods of Enterolobium cyclocarpum on fermentation, methane emissions and rumen microbial population in crossbred heifers. Anim. Feed. Sci. Technol. 251, 1–11. doi: 10.1016/j.anifeedsci.2019.01.011

[B47] Jacek D. Barbara M. Marta K. L. Marcin G. Janusz M. (2016). Pantoea agglomerans: A mysterious bacterium of evil and good. Part III. Deleterious effects: Infections of humans, animals and plants. Ann. Agr. Env. Med. 23 (2), 197–205. doi: 10.5604/12321966.1203878 27294620

[B48] Janardhan G. Gowda S. T. Thippesh D. Mahanthesh B. Ramachandra C. (2008). Effect of integrated nitrogen management practices on nutrient content of mulberry (Morus indica L.) leaves. Res. Crop 9 (2), 331–334.

[B49] Janda J. M. Abbott S. L. Cheung W. K. Hanson D. F. (1994). Biochemical identification of citrobacteria in the clinical laboratory. J. Clin. Microbiol. 32 (8), 1850–1854. doi: 10.1128/jcm.32.8.1850-1854.1994 7989531 PMC263890

[B50] Jitjaicham M. Kusuktham B. (2016). Preparation of paper mulberry fibers and possibility of cotton/paper mulberry yarns production. Ind. J. Mater. Sci. 2016, 1–6. doi: 10.1155/2016/1498967

[B51] Kagya-Agyemang J. Takyi-Boampong G. Adjei M. Karikari-Bonsu F. (2007). A note on the effect of Gliricidia sepium leaf meal on the growth performance and carcass characteristics of broiler chickens. J. Anim. Feed. Sci. 16 (1), 104–108. doi: 10.22358/jafs/66731/2007

[B52] Kandylis K. Hadjigeorgiou I. Harizanis P. (2009). The nutritive value of mulberry leaves (Morus alba) as a feed supplement for sheep. Trop. Anim. Health Pro. 41 (1), 17–24. doi: 10.1007/s11250-008-9149-y 19052898

[B53] Ke W. C. Ding W. R. Xu D. M. Ding L. M. Zhang P. Li F. D.. (2017). Effects of addition of Malic or citric acids on fermentation quality and chemical characteristics of alfalfa silage. J. Dairy. Sci. 100 (11), 8958–8966. doi: 10.3168/jds.2017-12875 28918135

[B54] Keeling L. Tunon H. Olmos A. G. Berg C. Jones M. Stuardo L.. (2019). Animal welfare and the united nations sustainable development goals. Front. Vet. Sci. 6. doi: 10.3389/fvets.2019.00336 PMC6797006 31649940

[B55] Kholif A. E. Gouda G. A. Anele U. Y. Galyean M. L. (2018). Extract of Moringa oleifera leaves improves feed utilization of lactating Nubian goats. Small. Ruminant. Res. 158, 69–75. doi: 10.1016/j.smallrumres.2017.10.014

[B56] Komarek A. M. Dunston S. Enahoro D. Godfray H. Herrero M. Mason-D’Croz D.. (2021). Income, consumer preferences, and the future of livestock-derived food demand. Global Environ. Change 70, 102343. doi: 10.1016/j.gloenvcha.2021.102343 PMC7612057 34857999

[B57] Kong L. Yang C. Dong L. Diao Q. Si B. Ma J.. (2019). Rumen fermentation characteristics in pre- and post-weaning calves upon feeding with mulberry Leaf flavonoids and candida tropicalis individually or in combination as a supplement. Animals 9 (11), 990. doi: 10.3390/ani9110990 31752155 PMC6912756

[B58] Leone A. Spada A. Battezzati A. Schiraldi A. Aristil J. Bertoli S. (2015). Cultivation, genetic, ethnopharmacology, phytochemistry and pharmacology of moringa oleifera leaves: An overview. Int. J. Mol. Sci. 16 (6), 12791–12835. doi: 10.3390/ijms160612791 26057747 PMC4490473

[B59] Li R. Jiang D. Zheng M. Tian P. Zheng M. Xu C. (2020). Microbial community dynamics during alfalfa silage with or without clostridial fermentation. Sci. Rep-UK. 10 (1), 17782. doi: 10.1038/s41598-020-74958-1 PMC7576192 33082504

[B60] Li Y. Nishino N. (2013). Effects of ensiling fermentation and aerobic deterioration on the bacterial community in Italian ryegrass, Guinea grass, and whole-crop maize silages stored at high moisture content. Asian-Austral. J. Anim. Sci. 26, 1304–1312. doi: 10.5713/ajas.2013.13175 PMC4093406 25049913

[B61] Lim T. K. (2014). “Gliricidia sepium,” in Edible medicinal and non-medicinal plants (Dordrecht: Springer), 806–816.

[B62] Liu Q. Dong Z. Shao T. (2018). Effect of additives on fatty acid profile of high moisture alfalfa silage during ensiling and after exposure to air. Anim. Feed. Sci. Technol. 236, 29–38. doi: 10.1016/j.anifeedsci.2017.11.022

[B63] Liu Y. Xiao Y. Xie J. Peng Y. Li F. Chen C.. (2022). Dietary supplementation with flavonoids from mulberry leaves improves growth performance and meat quality, and alters lipid metabolism of skeletal muscle in a Chinese Hybrid pig. Anim. Feed. Sci. Technol. 285, 115211. doi: 10.1016/j.anifeedsci.2022.115211

[B64] Ma G. Chai X. Hou G. Zhao F. Meng Q. (2022). Phytochemistry, bioactivities and future prospects of mulberry leaves: A review. Food Chem. 372, 131335. doi: 10.1016/j.foodchem.2021.131335 34818743

[B65] Maasdorp B. V. Muchenje V. Titterton M. (1999). Palatability and effect on dairy cow milk yield of dried fodder from the forage trees Acacia Boliviana, Calliandra calothyrsus and Leucaena leucocephala. Anim. Feed. Sci. Technol. 77 (1-2), 49–59. doi: 10.1016/S0377-8401(98)00232-6

[B66] Madhav P. N. Carolyn J. F. (2012). Phylogenetics of Morus (Moraceae) inferred from its and trnL-trnF sequence data. Syst. Bot. 37, 442–450. doi: 10.2307/41515134

[B67] Maqsood M. Saeed A. R. Sahar A. Khan I. M. (2022). Mulberry plant as a source of functional food with therapeutic and nutritional applications: A review. J. Food Biochem. 46 (11), e14263. doi: 10.1111/jfbc.14263 35642132

[B68] Marsetyo Sulendre I. W. Takdir M. Harper K. J. Poppi D. P. (2021). Formulating diets based on whole cassava tuber (Manihot esculenta) and gliricidia (Gliricidia sepium) increased feed intake, liveweight gain and income over feed cost of Ongole and Bali bulls fed low quality forage in Central Sulawesi, Indonesia. Anim. Prod. Sci. 61 (8), 761–769. doi: 10.1071/AN20297

[B69] Martínez M. Motta W. Cervera C. Pla M. (2005). Feeding mulberry leaves to fattening rabbits: effects on growth, carcass characteristics and meat quality. Anim. Sci. 80 (3), 275–280. doi: 10.1079/ASC41110275

[B70] McDonald P. Henderson A. R. Heron S. J. E. (1991). The biochemistry of silage. 2nd ed. (Marlow, UK: Chalcombe Publications).

[B71] Méndez-García C. Peláez A. I. Mesa V. Sánchez J. Golyshina O. V. Ferrer M. (2015). Microbial diversity and metabolic networks in acid mine drainage habitats. Front. Microbiol. 6. doi: 10.3389/fmicb.2015.00475 PMC4448039 26074887

[B72] Mpairwe D. R. Sabiiti E. N. Mugerwa J. S. (1998). Effect of dried Gliricidia sepium leaf supplement on feed intake, digestibility and nitrogen retention in sheep fed dried KW4 elephant grass (Pennisetum purpureum) ad libitum. Agroforest. Syst. 41 (2), 139–150. doi: 10.1023/A:1006097902270

[B73] Muck R. E. Nadeau E. M. G. McAllister T. A. Contreras-Govea F. E. Santos M. C. Kung L. (2018). Silage review: Recent advances and future uses of silage additives. J. Dairy. Sci. 101, 3980–4000. doi: 10.3168/jds.2017-13839 29685273

[B74] Mukumbo F. E. Maphosa V. Hugo A. Nkukwana T. T. Mabusela T. P. Muchenje V. (2014). Effect of Moringa oleifera leaf meal on finisher pig growth performance, meat quality, shelf life and fatty acid composition of pork. S. Afr. J. Anim. Sci. 44 (4), 388–400. doi: 10.4314/sajas.v44i4.9

[B75] Nallathambi Gunaseelan V. (1988). Anaerobic digestion of gliricidia leaves for biogas and organic manure. Biomass 17 (1), 1–11. doi: 10.1016/0144-4565(88)90066-2

[B76] Okoye C. O. Wang Y. Gao L. Wu Y. Li X. Sun J.. (2022). The performance of lactic acid bacteria in silage production: A review of modern biotechnology for silage improvement. Microbiol. Res. 266, 127212. doi: 10.1016/j.micres.2022.127212 36240665

[B77] Oliveira A. P. D. Bagaldo A. R. Loures D. R. S. Bezerra L. R. Moraes S. A. Yamamoto S. M.. (2018). Effect of ensiling gliricidia with cassava on silage quality, growth performance, digestibility, ingestive behavior and carcass traits in lambs. Anim. Feed. Sci. Technol. 241, 198–209. doi: 10.1016/j.anifeedsci.2018.05.004

[B78] Olugbenga D. O. Johnson O. A. Simeon O. A. Eyanlola S. A. Olajumoke T. D. Deborah A. O. (2018). Gliricidia leaf meal and multi-enzyme in rabbits diet: Effect on performance, blood indices, serum metabolites and antioxidant status. J. Anim. Sci. Technol. 60, 24. doi: 10.1186/s40781-018-0182-8 30323944 PMC6173935

[B79] Owen-Smith N. Cooper S. M. (1987). Palatability of woody plants to browsing ruminants in a South African Savanna. Ecology 2, 319–331. doi: 10.2307/1939263

[B80] Pagano C. Perioli L. Baiocchi C. Bartoccini A. Beccari T. Blasi F.. (2020). Preparation and characterization of polymeric microparticles loaded with Moringa oleifera leaf extract for exuding wound treatment. Int. J. Pharmaceut. 587, 119700. doi: 10.1016/j.ijpharm.2020.119700 32738457

[B81] Pakade V. Cukrowska E. Chimuka L. (2013). Metal and flavonol contents of *Moringa oleifera* grown in South Africa. S. Afr. J. Sci. 109, 3–4. doi: 10.1590/sajs.2013/835

[B82] Peñailillo J. Olivares G. Moncada X. Payacán C. Chang C. Chung K.. (2016). Correction: Sex distribution of paper mulberry (Broussonetia papyrifera) in the Pacific. PloS One 11 (9), e0163188. doi: 10.1371/journal.pone.0163188 27626936 PMC5023188

[B83] Phesatcha K. Wanapat M. (2016). Improvement of nutritive value and ruminal fermentation of silage by molasses and urea supplementation. Asian-. Asian-Austral. J. Anim. Sci. 29, 1136–1144. doi: 10.5713/ajas.15.0591 PMC4932567 26954159

[B84] Phimphachanhvongsod V. Ledin I. (2002). Performance of growing goats fed panicum maximum and leaves of gliricidia sepium. Asian-Austral. J. Anim. Sci. 15 (11), 1585–1590. doi: 10.5713/ajas.2002.1585

[B85] Rengsirikul K. Kanjanakuha A. Ishii Y. Kangvansaichol K. Sripichitt P. Punsuvon V.. (2011). Potential forage and biomass production of newly introduced varieties of leucaena (Leucaena leucocephala (Lam.) de Wit.) in Thailand. Grassl. Sci. 57, 94–100. doi: 10.1111/j.1744-697X.2011.00213.x

[B86] Roberson E. B. Firestone M. K. (1992). Relationship between desiccation and exopolysaccharide production in a soil Pseudomonas sp. Appl. Environ. Microb. 58 (4), 1284–1291. doi: 10.1128/aem.58.4.1284-1291.1992 PMC195588 16348695

[B87] Salem H. B. Makkar H. P. S. (2008). Defatted Moringa oleifera seed meal as a feed additive for sheep. Anim. Feed. Sci. Technol. 150 (1), 27–33. doi: 10.1016/j.anifeedsci.2008.07.007

[B88] Santos-Ricalde R. Gutiérrez-Ruiz E. Novelo-Ucan W. Martinez-Romero P. Segura-Correa J. (2017). Effect of feed restriction on intake of Moringa oleifera and Leucaena leucocephala and growth performance of rabbits. Trop. Anim. Health Pro. 49 (8), 1685–1688. doi: 10.1007/s11250-017-1377-6 28804858

[B89] Sheng P. He L. Ji S. Huang J. Zhang Z. Wang D.. (2021). Effect of Broussonetia papyrifera L. (Paper mulberry) on growth performance, carcase traits, meat quality and immune performance in Hu ram lambs. Ital. J. Anim. Sci. 20 (1), 691–697. doi: 10.1080/1828051X.2021.1904795

[B90] Si B. Tao H. Zhang X. Guo J. Cui K. Tu Y.. (2018). Effect of Broussonetia papyrifera L. (paper mulberry) silage on dry matter intake, milk composition, antioxidant capacity and milk fatty acid profile in dairy cows. Asian-Austral. J. Anim. Sci. 31 (8), 1259–1266. doi: 10.5713/ajas.17.0847 PMC6043428 29381894

[B91] Si Q. Wang Z. Liu W. Liu M. Ge G. Jia Y.. (2023). Influence of cellulase or Lactiplantibacillus plantarum on the ensiling performance and bacterial community in mixed silage of alfalfa and leymus chinensis. Microorganisms 11 (2), 426. doi: 10.3390/microorganisms11020426 36838391 PMC9964000

[B92] Singh R. Bhatia D. Bhasin V. (1997). Evaluation of paper mulberry (Broussonetia papyrifera) leaves in adult rabbits. Indian J. Anim. Sci. 67, 170–171.

[B93] Soedarjo M. Borthakur D. (1996). Simple procedures to remove mimosine from young leaves, pods and seeds of Leucaena leucocephala used as food. Int. J. Food Sci. Tech. 31 (1), 97–103. doi: 10.1111/j.1365-2621.1996.24-321.x

[B94] Song W. T. Zhu F. F. Chen K. P. (2021). The molecular mechanisms and factors affecting the feeding habits of silkworm (Lepidoptera: Bombyxidae). J. Asia-Pac. Entomol. 24 (4), 955–962. doi: 10.1016/j.aspen.2021.08.010

[B95] Srinivasulu C. Reddy M. Reddy D. (1999). Effect of supplementation of Gliricidia maculata to urea-treated and untreated paddy straw in crossbred cows. Indian J. Anim. Sci. 69 (5), 357–359.

[B96] Su B. Chen X. (2020). Current status and potential of moringa oleifera leaf as an alternative protein source for animal feeds. Front. Vet. Sci. 7. doi: 10.3389/fvets.2020.00053 PMC7054280 32175333

[B97] Takasaki M. Ogura R. Morikawa H. Chino S. Tsuiki H. (2011). Preparation and properties of paper yarn from mulberry. Adv. Mat. Res. 175-176, 575–579. doi: 10.4028/www.scientific.net/AMR.175-176.575

[B98] Tambone F. Pradella M. Bedussi F. Adani F. (2020). *Moringa oleifera* Lam. as an energy crop for biogas production in developing countries. Biomass Convers. Bior. 10, 1083–1089. doi: 10.1007/s13399-019-00550-x

[B99] Tang T. Bai J. Ao Z. Wei Z. Hu Y. Liu S. (2021). Effects of dietary paper mulberry (Broussonetia papyrifera) on growth performance and muscle quality of grass carp (ctenopharyngodon idella). Animals 11 (6), 1655. doi: 10.3390/ani11061655 34199491 PMC8227960

[B100] Tao H. Si B. Xu W. Tu Y. Diao Q. (2020). Effect of Broussonetia papyrifera L. Silage on blood biochemical parameters, growth performance, meat amino acids and fatty acids compositions in beef cattle. Asian-Austral. J. Anim. Sci. 33, 732–741. doi: 10.5713/ajas.19.0150 PMC7206392 32054236

[B101] Thompson L. J. Gray V. M. Kalala B. Lindsay D. Reynolds K. von Holy A. (2008). Biohydrogen production by Enterobacter cloacae and Citrobacter freundii in carrier induced granules. Biotechnol. Lett. 30 (2), 271–274. doi: 10.1007/s10529-007-9527-y 17876534

[B102] Tian M. Wang Y. Lu A. Zhang Q. Li X. Zhang N.. (2022). From metabolomic analysis to quality assessment and biosynthetic insight in traditional Chinese medicine: Mulberry tree as a case study. Phytochem. Anal. 33 (4), 644–653. doi: 10.1002/pca.3117 35233869

[B103] Tudsri S. Chotchutima S. Nakamanee K. Kangwansaichol K. (2019). Dual use of leucaena for bioenergy and animal feed in Thailand. Trop. Grassl-Forrajes. 7 (2), 193–199. doi: 10.17138/tgft(7)193-199

[B104] Uzal F. A. Navarro M. A. Li J. Freedman J. C. Shrestha A. McClane B. A. (2018). Comparative pathogenesis of enteric clostridial infections in humans and animals. Anaerobe 53, 11–20. doi: 10.1016/j.anaerobe.2018.06.002 29883627 PMC6281819

[B105] Vandermeulen S. Ramírez-Restrepo C. A. Beckers Y. Claessens H. Bindelle J. (2017). Agroforestry for ruminants: A review of trees and shrubs as fodder in silvopastoral temperate and tropical production systems. Anim. Prod. Sci. 58 (5), 767–777. doi: 10.1071/AN16434

[B106] Wang C. Liu Q. Meng J. Yang W. Yang X. He D.. (2009). Effects of citric acid supplementation on rumen fermentation, urinary excretion of purine derivatives and feed digestibility in steers. J. Sci. Food Agr. 89 (13), 2302–2307. doi: 10.1002/jsfa.3724

[B107] Wang H. Ning T. Hao W. Zheng M. Xu C. (2016). Dynamics associated with prolonged ensiling and aerobic deterioration of total mixed ration silage containing whole crop corn. Asian-Austral. J. Anim. Sci. 29 (1), 62–72. doi: 10.5713/ajas.15.0319 PMC4698690 26732329

[B108] Wang Z. Ning P. Hu L. Nie Q. Liu Y. Zhou Y.. (2020). Efficient ethanol production from paper mulberry pretreated at high solid loading in fed-nonisothermal-simultaneous saccharification and fermentation. Renew. Energ. 160, 211–219. doi: 10.1016/j.renene.2020.06.128

[B109] Wang Y. Wu K. Zhen Z. Shi Z. Yang H. Zhang J.. (2018). Combined pretreatment with NaOH and fenton of mulberry wood to enhance enzymatic digestibility for biofuels. J. Biobased. Mater. Bio. 12 (1), 65–75. doi: 10.1166/jbmb.2018.1734

[B110] Wang Y. Zhang Y. Li J. Lin J. Zhang N. Cao W. (2021). Biogas energy generated from livestock manure in China: Current situation and future trends. J. Environ. Manage. 297, 113324. doi: 10.1016/j.jenvman.2021.113324 34298348

[B111] Wen P. Hu T. Linhardt R. J. Liao S. Wu H. Zou Y. (2018). Mulberry: A review of bioactive compounds and advanced processing technology. Trends Food Sci. Tech. 83, 138–158. doi: 10.1016/j.tifs.2018.11.017

[B112] Wu Z. Liang C. Huang R. Ouyang J. Zhao L. Bu D. (2022). Replacing alfalfa hay with paper mulberry (Broussonetia papyrifera L.) silage in diets do not affect the production performance of the low lactating dairy cows. Anim. Feed. Sci. Technol. 294, 115477. doi: 10.1016/j.anifeedsci.2022.115477

[B113] Wu Y. Xu Y. Lau A. T. Y. (2021). Anti-cancer and medicinal potentials of moringa isothiocyanate. Molecules 26 (24), 7512. doi: 10.3390/molecules26247512 34946594 PMC8708952

[B114] Xiong Y. Guo C. Wang L. Chen F. Dong X. Li X.. (2021). Effects of paper mulberry silage on the growth performance, rumen microbiota and muscle fatty acid composition in Hu lambs. Fermentation 7 (4), 286. doi: 10.3390/fermentation7040286

[B115] Yang S. L. Yang R. C. Zhou X. Yang S. H. Luo L. L. Zhu Y. C.. (2020). Effects of feeding diets with processed Moringa oleifera stem meal on growth and laying performance, and immunological and antioxidant activities in laying ducks. Poultry. Sci. 99 (7), 3445–3451. doi: 10.1016/j.psj.2020.04.002 32616238 PMC7597767

[B116] Zhang Y. C. Li D. X. Wang X. K. Lin Y. L. Zhang Q. Chen X. Y.. (2019). Fermentation dynamics and diversity of bacterial community in four typical woody forages. Ann. Microbiol. 69 (3), 233–240. doi: 10.1007/s13213-018-1398-z

[B117] Zhang Y. Ni S. Wu R. Fu Y. Qin M. Willför S.. (2022). Green fractionation approaches for isolation of biopolymers and the critical technical challenges. Ind. Crops. Prod. 177 (4), 114451. doi: 10.1016/j.indcrop.2021.114451

[B118] Zhang Y. C. Wang X. K. Li D. X. Lin Y. L. Yang F. Y. Ni K. K. (2020). Impact of wilting and additives on fermentation quality and carbohydrate composition of mulberry silage. Asian-Austral. J. Anim. Sci. 33 (2), 254–263. doi: 10.5713/ajas.18.0925 PMC6946962 31208169

